# Exposing structural variations in SARS-CoV-2 evolution

**DOI:** 10.1038/s41598-021-01650-3

**Published:** 2021-11-11

**Authors:** Jiaan Yang, Peng Zhang, Wen Xiang Cheng, Youyong Lu, Wu Gang, Gang Ren

**Affiliations:** 1 Shenzhen Institutes of Advanced Technology, Chinese Academy of Sciences, Shenzhen, 518055 Guangdong China; 2 Micro Biotech, Ltd., Shanghai, 200123 China; 3 Laboratory of Molecular Oncology, Peking University Cancer Hospital and Institute, Beijing, 100142 China; 4 School of Basic Medicine, Tongji Medical College, Huazhong University of Science and Technology, Wuhan, 430030 China; 5 The Molecular Foundry, Lawrence Berkeley National Laboratory, Berkeley, CA 94720 USA

**Keywords:** Viral infection, Structural biology

## Abstract

The mutation of SARS-CoV-2 influences viral function as residue replacements affect both physiochemical properties and folding conformations. Although a large amount of data on SARS-CoV-2 is available, the investigation of how viral functions change in response to mutations is hampered by a lack of effective structural analysis. Here, we exploit the advances of protein structure fingerprint technology to study the folding conformational changes induced by mutations. With integration of both protein sequences and folding conformations, the structures are aligned for SARS-CoV to SARS-CoV-2, including Alpha variant (lineage B.1.1.7) and Delta variant (lineage B.1.617.2). The results showed that the virus evolution with change in mutational positions and physicochemical properties increased the affinity between spike protein and ACE2, which plays a critical role in coronavirus entry into human cells. Additionally, these structural variations impact vaccine effectiveness and drug function over the course of SARS-CoV-2 evolution. The analysis of structural variations revealed how the coronavirus has gradually evolved in both structure and function and how the SARS-CoV-2 variants have contributed to more severe acute disease worldwide.

## Introduction

Currently, there exists an urgent need to explore the structure, function and activity of the severe acute respiratory syndrome coronavirus 2 (SARS-CoV-2), belonging to the coronavirus family^1^. In particular, the study of mutations in SARS-CoV-2 is considered a priority because of their potential to increase transmissibility and virulence while reducing the effectiveness of vaccines and impacting the development of therapeutic drugs^2,3^. Mutations, that alter the protein sequence, including replacements or deletions of amino acid residues, may affect protein properties and folding conformations and result in changes to the biological functions of the virus^4^. The interaction of the receptor-binding domain (RBD) of the spike protein of SARS-CoV-2 with angiotensin-converting enzyme 2 (ACE2) receptors is key for allowing the virus to enter human cells^5,6^. Thus, the mutations in the RBD directly influence the epidemic coronavirus disease spread^7,8^.

To date, over 3000 of SARS-CoV-2 sequences and nearly 800 of 3D structures of spike protein data sources are available in National Center for Biotechnology Information (NCBI) database and Protein Data Bank (PDB). According to the COVID-19 Genomics UK (COG-UK) Consortium, more than 4000 mutations have been detected in the spike protein alone^9^, which provides sufficient data for investigation of coronavirus mutations, and helps to understand the changes in its physiochemical properties as well as folding conformations leading to virus evolution over time. With protein sequence alignment, the positions of replaced amino acid residues can be discovered, and the concomitant changes in physiochemical properties can be further probed^9,10^. In addition to physiochemical properties, the changes in protein folding conformation also impact biological viral functions. For proteins with known 3D structures, the conformational differences caused by mutations can be roughly compared by structure superposition with root-mean-square deviation (RMSD) as a measurement^11^. For proteins without known 3D structures, the protein structures first need to be predicted by computational dynamics simulations. However, for mutational differences, the reliability of the predicted protein structure remains a challenge even when using ab initio modeling methods^12–14^. Thus, it is crucial that a new approach overcomes these barriers to study structural mutations.

At this point, we propose using the protein structure fingerprint approach^15,16^ to analyze the changes in folding conformation caused by mutations. With protein structure fingerprints, the protein folding shape code (PFSC) provides an alphabetical string to completely describe the folding conformation for 3D protein structure. Additionally, according to the protein sequence, the acquired protein folding variation matrix (PFVM) reveals the folding variations along the sequence, and also it is able to generate the most possible folding conformations. Thus, the alignment of the protein sequence with the PFSC string can comprehensively expose the variations in both biological functions and folding conformations caused by mutations in SARS-CoV-2. Here, the structural variations in the evolved coronavirus strains, from SARS-CoV to SARS-CoV-2 including Alpha variant (lineage B.1.1.7) and Delta variant (lineage B.1.617.2), are studied.

## Results

The changes in both physiochemical properties and folding conformations of SARS-CoV-2 due to mutation are studied based on the 3D structures and sequences of the spike protein, and the interaction between coronavirus and ACE2 are a particular focus. The structural analysis covers coronavirus strains from early SARS-CoV to SARS-CoV-2 and its variants.

### Variations based on 3D structures

Coronavirus spike proteins have an S1 subunit at the N-terminus (~ 700 amino acids) and an S2 subunit at the C-terminus (~ 600 amino acids). Analysis of many protein 3D structures confirmed that three S1/S2 heterodimers assembled to form a trimer spike protruding from the viral envelope^17^. The S1 subunit of the spike protein in SARS-CoV-2 is an envelope glycoprotein that plays the most important role in viral attachment, fusion, and entry into host cells, and it is a major target for the development of neutralizing antibodies, inhibitors and vaccines. The S1 subunit contains a receptor-binding domain (RBD), and many studies have found that the RBD of the spike protein in SARS-CoV-2 strongly bound to human and bat angiotensin- converting enzyme 2 (ACE2) receptors^18–20^.

A set of sequences for the cluster of the SARS-CoV-like_Spike_S1_RBD subfamily (cd21477) that contains the conserved protein domain of the S1 RBD subfamily for SARS-CoV-like and SARS-CoV-2 spike proteins (with GenBank: APO40579.1) is available in the NCBI database. The sequences of the cd21477 cluster are aligned and presented in Table 1, where the red color font indicates highly conserved fragments, blue indicates less conserved fragments and gray indicates unaligned fragments. It is not surprising that the mutations were most frequent on less conserved residues (blue font). Additionally, it is noted that some sequences have the given 3D structures in the PDB. The protein structure of the SARS-CoV spike protein, with PDB ID 6ACC, was released in August 2018^21^; the protein structure of the SARS-CoV-2 spike protein, with PDB ID 6VSB, was released in February 2020^17^. The residues that differ between 6ACC and 6VSB are marked in green. Some changes in the physiochemical properties based on the residue differences between 6ACC to 6VSB are summarized in Table 2, including hydrophobicity, negative or positive charge, polarity, size of side chain, aromatic, etc. The changes in the physiochemical properties are represented by a “+” sign for an increase in the property after mutation and a “−” sign for a decrease in the property after mutation.

**Table 1:**
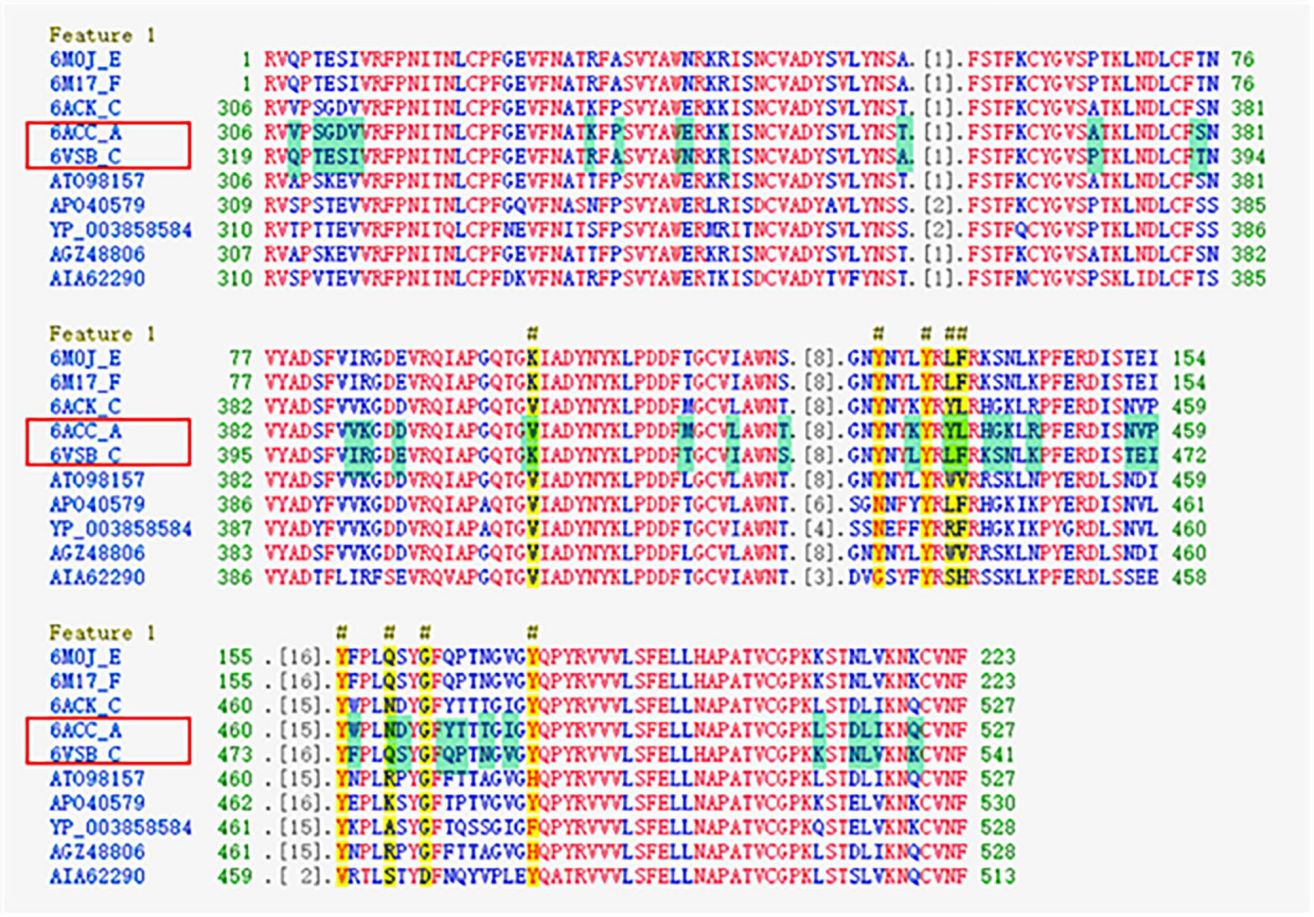
The protein domain conservation and variation for sequences of SARS-CoV-like_Spike_S1_RBD subfamily of cd21477 in NCBI. In sequence, red font indicates highly conserved, blue for less conserved and gray for unaligned as the threshold 3.5 for conservation alignment. Green background indicates amino acid differences between 6ACC-A for SARS-CoV and 6VSB-C for SARS-CoV-2.

**Table 2:**
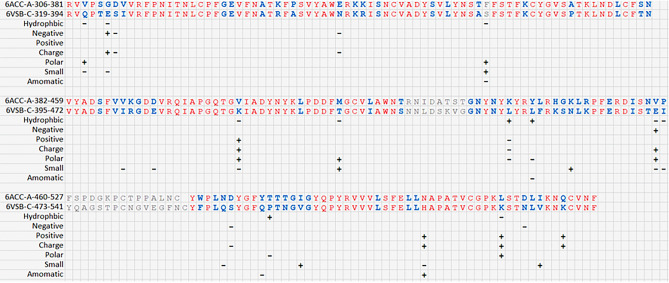
The change of physicochemical properties due to mutations from sequence of 6ACC for SARS-CoV to 6VSB for SARS-CoV-2. The two rows on top are sequences of 6ACC and 6VSB, and the residues in red are highly conserved, blue are less conserved and gray are unaligned. The physicochemical properties are listed in the left column. The “ + ” sign indicates an increase in the property after mutation; the “−” sign indicates a decrease in the property after mutation.

**Figure 1:**
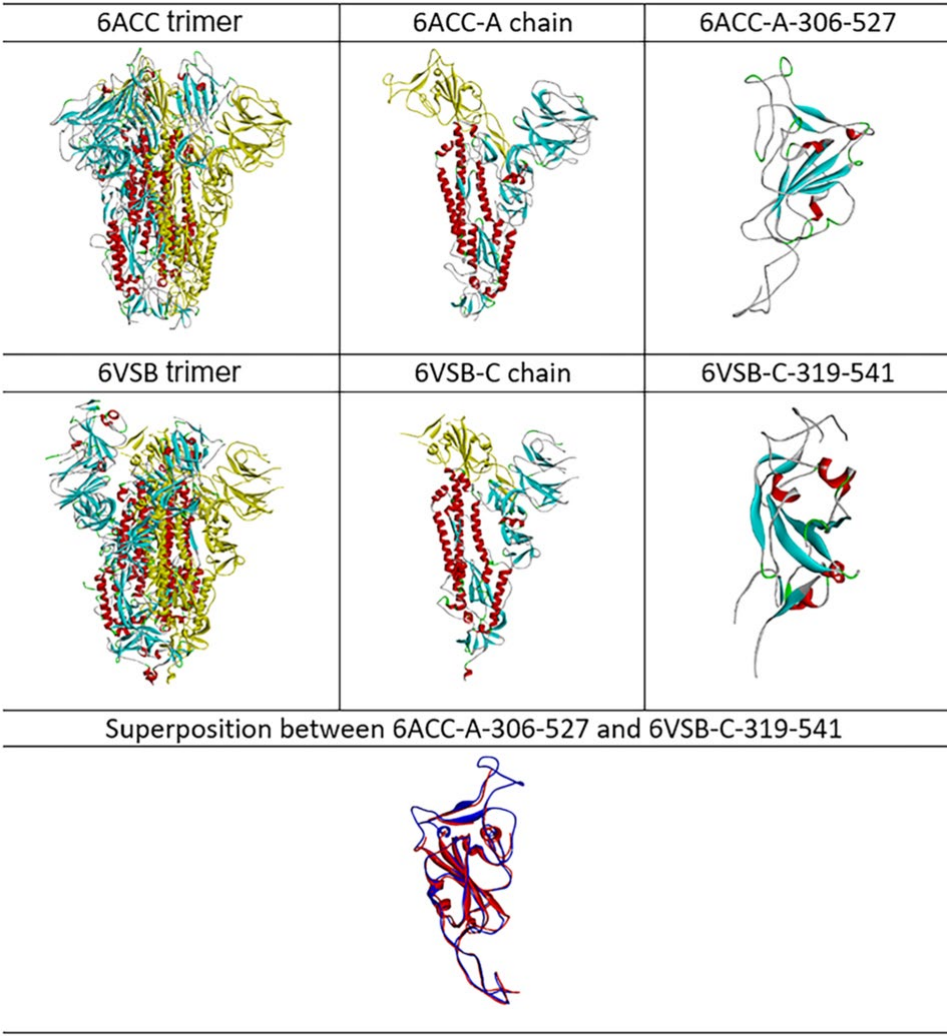
Comparison of 3D structures between 6ACC and 6VSB. The structure of PDB ID 6ACC is the SARS-CoV spike protein, and PDB ID 6VSB is the SARS-CoV-2 spike protein. The trimer of protein 3D structure, chain and domain fragment are displayed. The superposition of fragments between 6ACC-A-306-527 (blue) and 6VSB-C-319-541 (red) are shown at the bottom.

With the given structures of 6ACC and 6VSB, their 3D images of folding conformations are compared and displayed in Fig. 1. The 3D structures directly provide a visualization to observe the protein structures, and the superposition allows a comparison of the structures. Although more than 30 mutations in the fragment between 6ACC-A-306-527 for SARS-CoV and 6VSB-C-319-541 for SARS-CoV-2 occurred, representing up to 37.5% residue replacement, the structure superposition showed that the folding conformations of 6ACC and 6VSB were still similar overall. It is difficult to distinguish the folding differences of spike proteins between SARS-CoV and SARS-CoV-2 based on the 3D structure only. With the protein folding shape code (PFSC), however, the differences in folding can be exposed. Any protein 3D structure can be converted into a PFSC description, which is an alphabetical string representing the continuous folding shape of each five-amino-acid in sequence. Thus, the folding conformations of 6ACC for SARS-CoV and 6VSB for SARS-CoV-2 can be compared by PFSC alignment and displayed in Table 3. In PFSC, generally, the red color indicates typical alpha helix, pink indicates alpha-like helix, blue indicates a typical beta strand, light blue indicates a beta-like strand, and black indicates an irregular fold. According to the PFSC color notation, it is obvious that the secondary structural fragments are well aligned. For example, the fragments of alpha helices at 324–330 and 352–364 and the beta strands at 349–351 and 378–386 on 6ACC are aligned with the corresponding fragments in 6VSB. In addition, the PFSC alignment exposes local folding comparison in detail, in which the local folding similarity and differences between PFSC strings are indicated; “|” indicates an identical folding shape, “:” indicates a similar folding shape, and “.” indicates dissimilar folding. Thus, the changes of local folding conformations around the mutated residues can be exhibited. For example, the folding letters at 334, 335, 340, 341, 343, 370, 379, 380, 417, 426 and 515 on 6ACC are different from 6VSB. Also, due to mutations, the adjustments of beta strand at 349–351 and 452–454 fragments on 6ACC are exposed. Thus, the PFSC well revealed the changes in local folding shapes caused by the mutations.

**Table 3:**
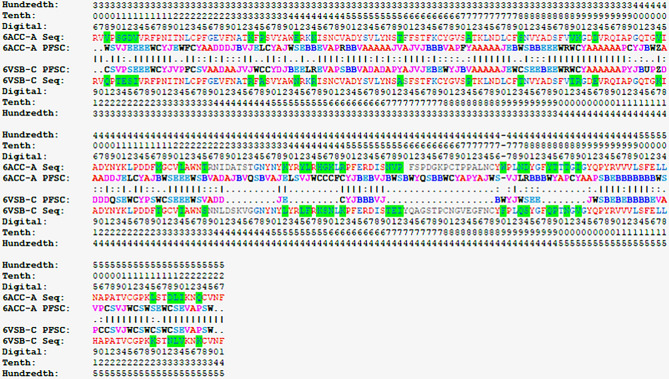
PFSC string alignment between SARS-CoV (PDB 6ACC-A-306–527) and SARS-CoV-2 (PDB 6VSB-C-319–541). The rule of residue position and amino acid sequences are above or below the sequence separately. In the sequence, red font indicates highly conserved, blue indicates less conserved and gray indicates unaligned. The green background indicates the different residues between two sequences. Each PFSC letter represents the folding shape of 5 amino acid residues. For PFSC, generally the red color indicates a typical alpha helix, pink indicates an alpha-like helix, blue indicates a typical beta strand, light blue indicates a beta-like strand, and black indicates an irregular fold. In the alignment, the local folding similarity and differences between PFSC strings are indicated; “|” indicates an identical folding shape, “:” indicates a similar folding shape, and “.” indicates dissimilar folding.

### Variations based on sequences

**Figure 2:**
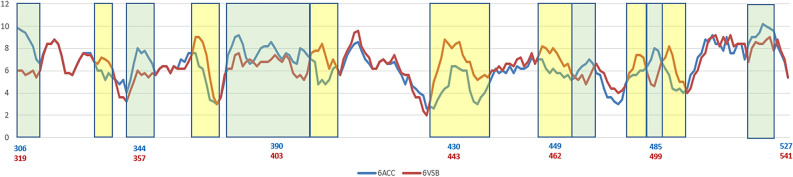
The numbers of folding variations in PFVM between 6ACC-A-306-527 for SARS-CoV and 6VSB-C-319-541 for SARS-CoV-2. The horizontal coordinate is the sequence position, and the longitudinal coordinate is the number of folding shapes, i.e., number of PFSC letters. Blue curve represents the change of numbers of folding variations along sequence for 6ACC; red curve for 6VSB. Yellow blocks indicate the ranges with more variation in SARS-CoV, whereas green blocks indicate more variation in SARS-CoV-2.

The variations of folding conformations for a protein based on sequence alone can be exposed by the PFVM. According to sequences taken directly from the structures of 6ACC-A-306-527 for SARS-CoV and 6VSB-C-319-541 for SARS-CoV-2 separately, the PFVMs are obtained and exhibited in Table 4. The PFSC letters in each column represent the folding variations of 5 successive amino acid residues in sequence while the favored folding shapes are ranked on top, and the numbers of PFSC letters are different in each column. The PFVM exhibits the folding variations along the sequence. The numeric deviations of folding shapes along sequences in PFVM between 6ACC-A-306-527 for SARS-CoV and 6VSB-C-319-541 for SARS-CoV-2 are shown by the curves in Fig. 2, in which the yellow and green blocks indicate the regions of fluctuation due to mutations. It was apparent that the mutations caused the changes in folding flexibility; some fragments potentially became more flexible, and other fragments are more rigid. Thus, along the sequence from the N-terminus to the C-terminus, the variations in the folding conformation are well exposed.

**Table 4:**
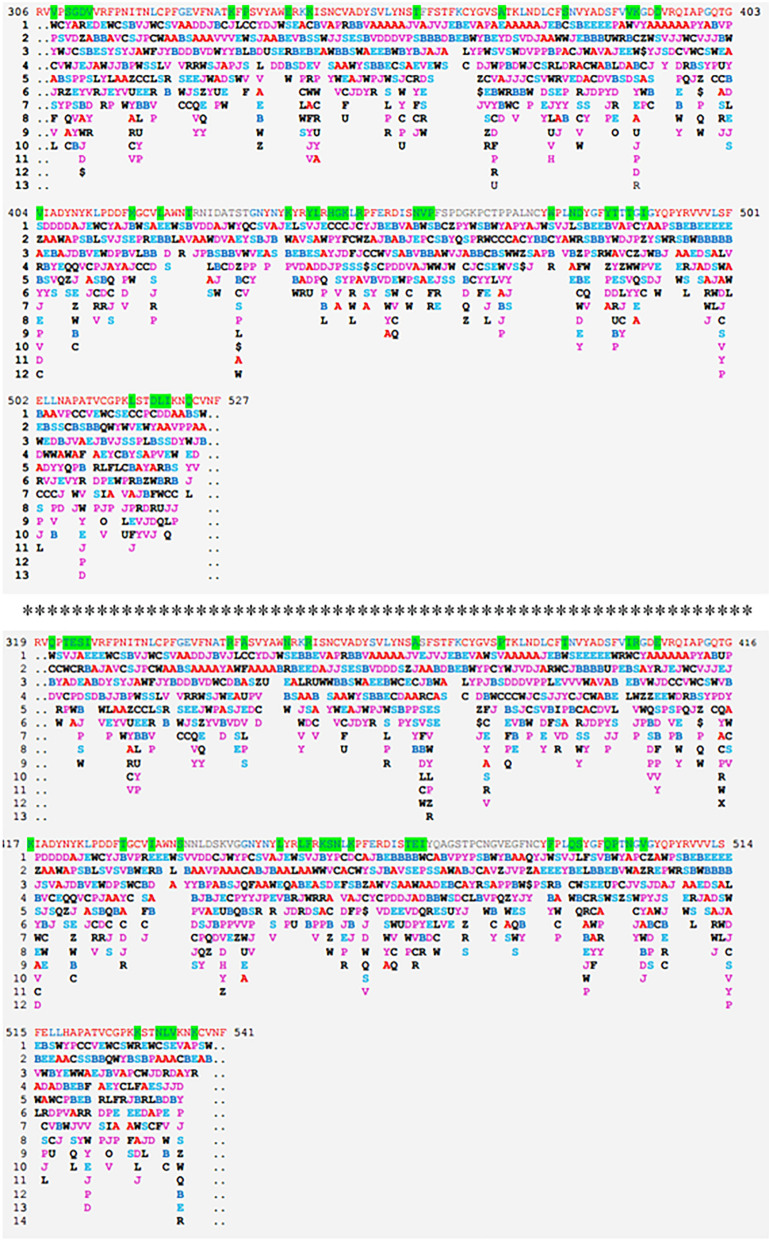
The protein folding variation matrix (PFVM). The PFVM on top was obtained according to the sequence for PDB 6ACC-A-306–527 for SARS-CoV; the PFVM on bottom represents for PDB 6VSB-C-319–541 for SARS-CoV-2. On each PFVM, the sequence is horizontally listed above matrix, and the PFSC letters in each column represent the folding variations of continuous 5 amino acid residues with the most favored folding shapes are on top. The PFVM displays the folding variations along the sequence from N-terminus to C-terminus.

The most likely conformations for a protein can be extracted from PFVM. Taking one letter from each column, a massive number of PFSC strings are formed, and each string represents one of possible folding conformations. Although a large number of folding conformations exist, the letters on top of each column are directly constructed into one folding string as the most likely conformations, which is named PFVM-01. This predicted conformation may be assessed by a given 3D protein structure through PFSC alignment. Two PFSC-01 strings for SARS-CoV and SARS-CoV-2, which are the folding strings at first row of PFVM from Table 4, and two PFSC strings, which are the folding conformations directly according to 3D structures of 6ACC-A-306-527 and 6VSB-C-319-541 from Table 3, are aligned and exhibited in Table 5. The PFSC letters in red and pink colors represent alpha helices, those in blue and light blue represent beta strands and those in black represent irregular folding shapes. Overall, with observation, the secondary fragments are aligned, so the predicted folding conformations of PFVM-01 for SARS-CoV and SARS-CoV-2 are similar to the given 3D structures. Thus, the PFVM-01 generated from PFVM is a reliable folding conformation according the sequence merely.

**Table 5:**
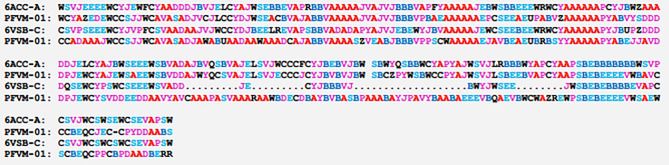
The alignment of PFSC strings between PDB 6ACC-A-306–527 for SARS-CoV and PDB 6VSB-C-319–541 for SARS-CoV-2, and the most likely folding conformation (PFVM-01) from PFVM. The left column indicates the structure names. Two of PFVM-01 are the PFSC strings taken from the first row of PFVM in Table 4. In PFSC letters, the red and pink represent alpha helix and like helical fold; the blue and light blue for beta strand and like beta strand.

### Structure variations with virus evolution

It is essential that the coronavirus evolution is analyzed with structural changes at molecule level. The sequence of the SARS-CoV spike protein (UniProtKB = P59594 (SPIKE_SARS)) was first determined in 2003^22^. The sequence of the SARS-CoV-2 spike protein (UniProtKB = P0DTC2 (SPIKE_SARS2)) was determined in January 2020^23^. After 17 years of evolution from SARS-CoV to SARS-CoV-2, the spike protein sequences are approximately 24% different. The Alpha variant (lineage B.1.1.7) is the mutant of SARS-CoV-2 that was noted in September 2020 from a sample taken in the UK in September, which increased infections in the UK because of one or more mutations in the virus spike protein. The Alpha variant (lineage B.1.1.7 VOC-202012/01) is taken from the Public Health England^24^, which was reported on March 5, 2021, with seven mutations in the spike protein: E484K, N501Y, A570D, P681H, T716I, S982A and D1118H^25^. Similar variants have also emerged in South Africa (lineage B.1.351) and Brazil (lineage P.1). Another Delta variant (lineage B.1.617.2 and sub-lineages AY.1 and AY.2) with mutations of K417N, N440K, L452R, T478K and E484Q in the spike protein was the first outbreak in India during October 2020, which caused the epidemic severe. Thus, it is important to understand the effects of the mutations following virus evolution.

In order to study the mutations of Alpha variant (lineage B.1.1.7) in SARS-CoV-2, a sequence of QTJ15692 (GenBank) was taken as the background reference, which was deposited in the NCBI database in April 2020 before the Alpha variant. The Alpha variant mutations may cause changes in physicochemical functions as well as in folding conformations, which together impact biological functions. The changes in physiochemical properties, including hydrophobicity, negative or positive charge, polarity, residue size and aromaticity, are listed in the top seven rows of Table 6. For example, the mutation E484K is a change from a negative charge to a positive charge; A570D is a change from hydrophobic to hydrophilic, from non-charge to negative charge and from non-polar to polar; P681H is a change from hydrophobic to hydrophilic, from non-polar to polar and an increase in the size of the side chain due to an aromatic moiety; T716I and S982A are changes from polar to non-polar and from hydrophilic to hydrophobic. Thus the changes in physiochemical properties caused by mutations are indicated in detail. Furthermore, the variations in local folding shapes may be revealed by PFVM because each PFSC letter in PFVM represents the folding shape of 5 successive amino acids in sequence. The PFVMs of seven regions for these related mutations are displayed in Table 7, which shows the folding variations before and after mutations. To compare each pair of PFVMs, the fluctuations in the number of folding shapes and the contributions of the alpha helix and beta strand are summed and listed in the bottom three rows in Table 6. It is apparent that the number of folding variations is reduced after mutation for seven regions, indicating that the flexibilities are reduced. For the E484K mutation, the contribution of the alpha helix increased while the beta strand decreased; for N501Y, the contribution of the alpha helix decreased while the beta strand increased; for D1118H, the factor of contribution of the alpha helix decreased while the beta strand increased. Therefore, the variations in both physiochemical properties and folding features for Alpha variant (lineage B.1.1.7) mutations of SARS-CoV-2 are well exposed.

**Table 6:**
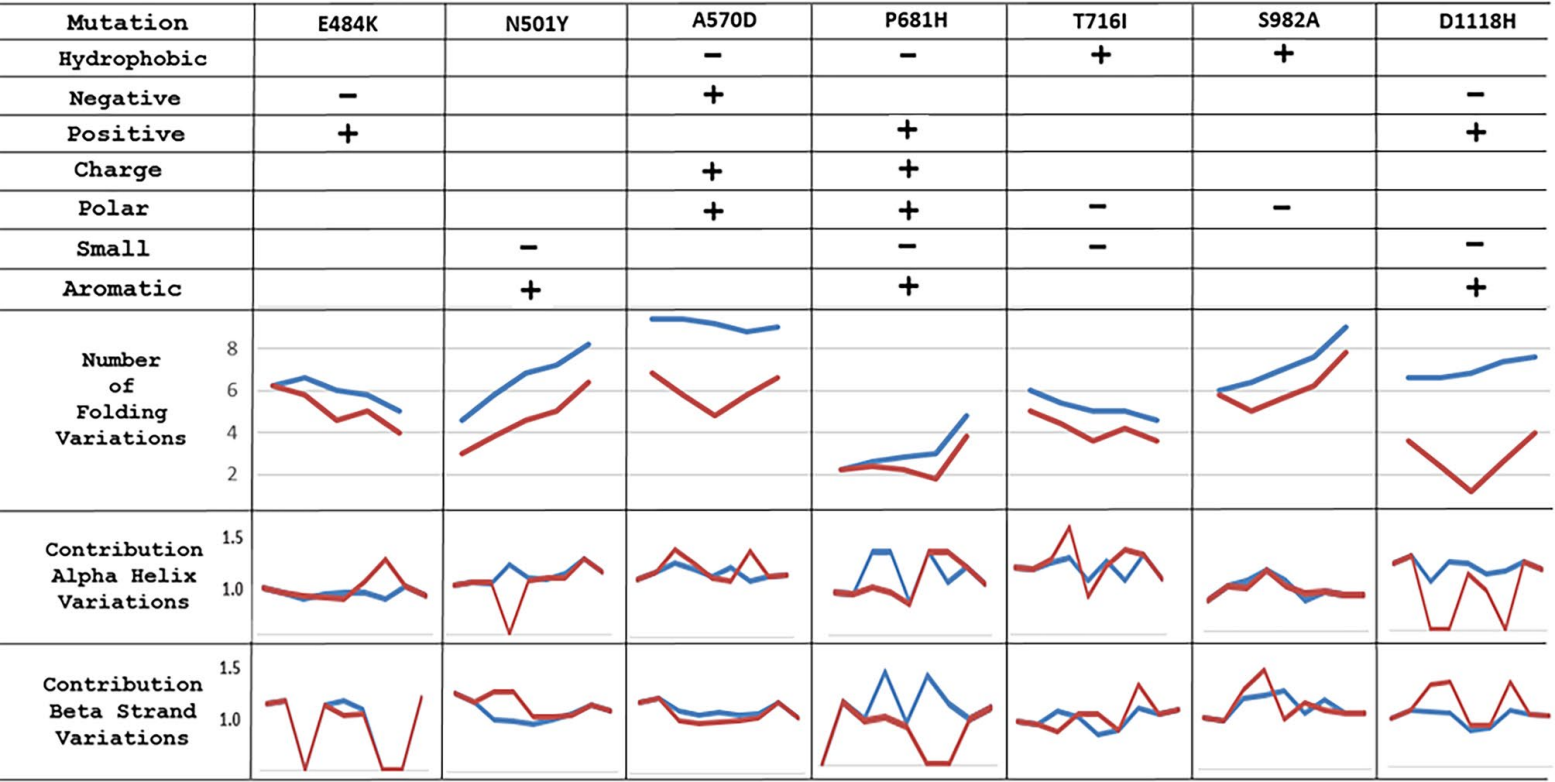
The variations of physiochemical properties and folding features for Alpha variant. Alpha variant is compared with GenBank QTJ15692.1 of SARS-CoV-2 as reference. The potential changes of physiochemical properties are listed on top seven rows, the “−” means a specific property decreased after mutation and the “+” property increased after mutation. The folding variations are listed at bottom three rows. The red curves represented the status after mutations; blue curves for status before mutations.

**Table 7:**
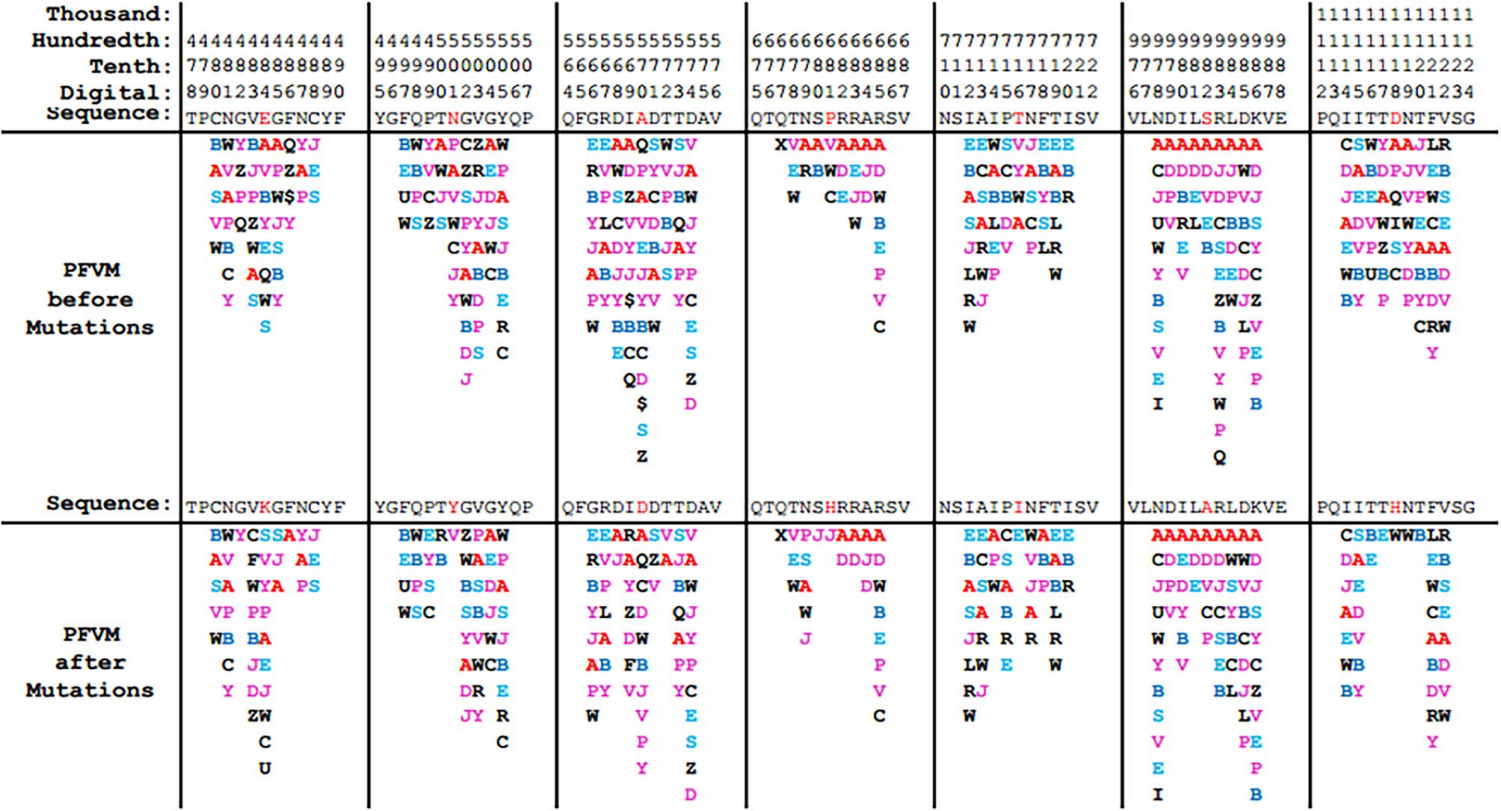
Comparison for sections of PFVM between before and after Alpha variant. The PFVM on top is for GenBank QTJ15692.1, and the PFVM on bottom is for the Alpha variant (E484K, N501Y, A570D, P681H, T716I, S982A and D1118H). The rule for the residue position is at the top, the sequences are listed above the PFVM, and the mutated residues are shown in red. In PFVM, the PFSC letters in each column represent the folding variations of 5 continue amino acid residues, and the most favored folding shapes are on top.

### Mutations versus ACE2 interaction

**Figure 3:**
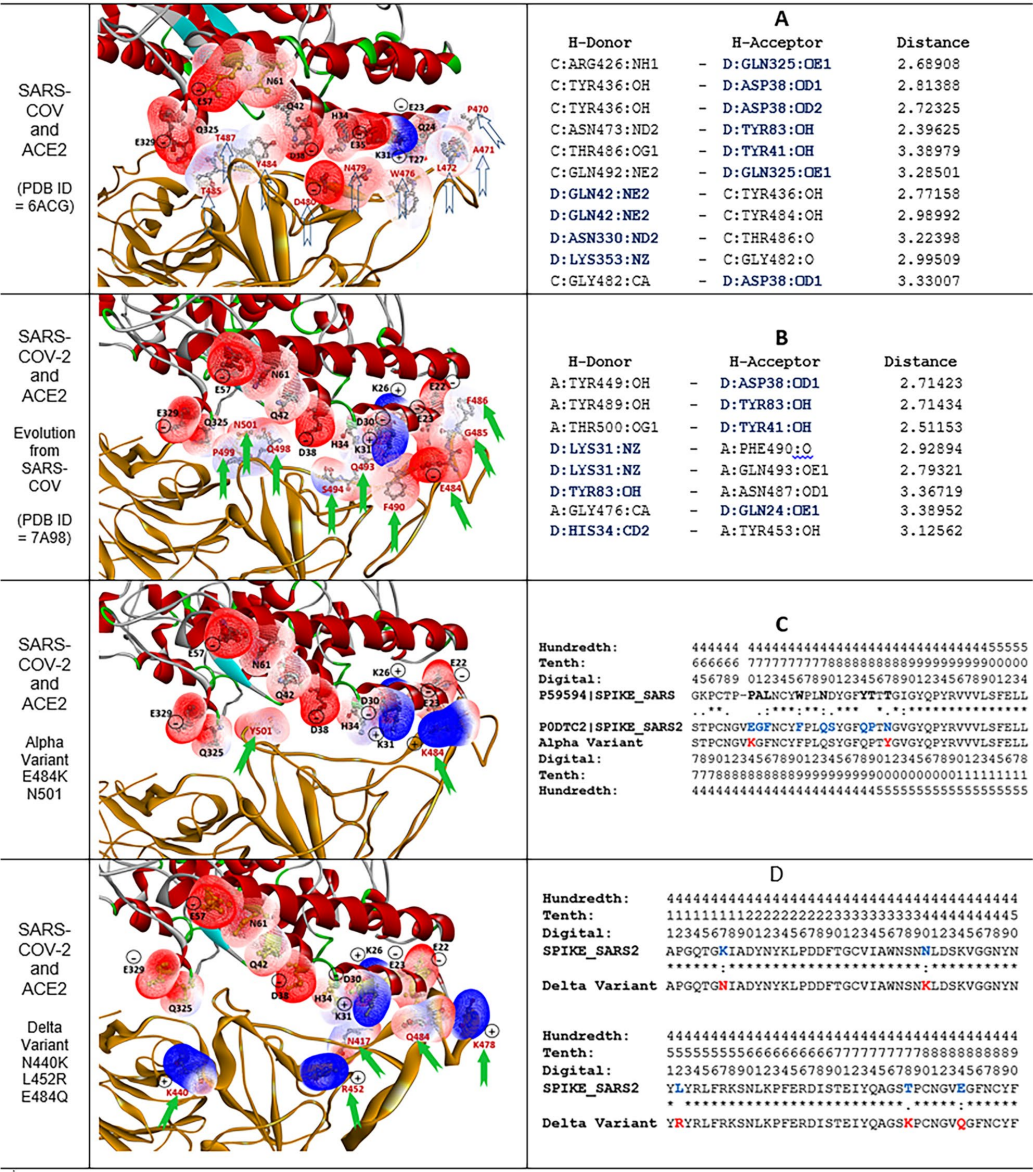
Coronavirus evolution enhanced the interaction of the spike protein with ACE2. The 3D images display the binding interface between RBD of the SARS-CoV-2 spike protein and ACE2. The protein structure shown in brown color is SARS-CoV or SARS-CoV-2. The wire meshes represent the charge surfaces for residues involved in the interaction; red wire mesh indicates negative charge, and blue indicates positive charge. Row **A** shows the SARS-CoV structure and intermolecular H-bonds (PDB ID 6ACG); row **B** shows SARS-CoV-2 and H-bonds (PDB ID 7A98). Row **C** shows the structure of the Alpha variant, and row **D** shows the structure of the Delta variant, which were both obtained by computational modeling. The contributions of hydrogen bonds from ACE2 are marked by blue bold font. The arrows indicate the residues altered in viral evolution. The sequences of SARS-CoV (UniProtKB = P59594 (SPIKE_SARS)), SARS-CoV-2 (UniProtKB = P0DTC2 (SPIKE_SARS2)) and the Alpha variant and Delta variant are aligned and listed in row **C** and **D**, and the mutational residues are shown in colored bold font.

The RBD of the spike protein of SARS-CoV-2 binds to angiotensin-converting enzyme 2 (ACE2) receptors, serving as the entry point into human cells and causing the global coronavirus disease pandemic^26^. Thus, analysis of mutations at the RBD of the SARS-CoV-2 spike protein is important for understanding the change of affinity with ACE2, which can explain why coronavirus became more dramatically widespread. The structure variation at the RBD fragment, as the interface affinitive with ACE2, is focused on in this study, especially the evolution from SARS-CoV to SARS-CoV-2, to Alpha variant (lineage B.1.1.7) and Delta variant (lineage B.1.617.2). The complete sequences of the spike proteins were obtained from the Universal Protein Resource (UniProt) database^27^, with UniProtKB P59594 for SARS-CoV (SPIKE_SARS) and UniProtKB P0DTC2 for SARS-CoV-2 (SPIKE_SARS2). Then, the mutational fragments of RBD sequences interfacing with ACE2 were aligned, and the evolution of sequences from SARS-CoV to SARS-CoV-2 and Alpha variant is displayed in Fig. 3C and the evolution from SARS-CoV-2 to Delta variant is displayed in Fig. 3D. The residues involving mutations are marked with bold font, in which SARS-CoV is black, SARS-CoV-2 blue, and Alpha variant and Delta variant red. Sequence alignment showed that the evolution of the RBD from SARS-CoV to SARS-CoV-2 involved the replacement of 9 residues; to the Alpha variant two residues; and to the Delta variant five residues.

The protein 3D structures of the complex of the spike protein and ACE2 are available in the PDB, and images of the interaction between the spike protein and ACE2 are displayed in Fig. 3. The protein structure with PDB ID 6ACG is the complex of the SARS-CoV spike protein and ACE2, which was deposited in July 2018; the structure with PDB ID 7A98 is the complex of the SARS-CoV-2 spike protein with ACE2, which was deposited in September 2020. The mutational residues of SARS-CoV-2 as well as the residues of ACE2 on the binding interface are marked by wire mesh to show the interpolated charged surface. It is apparent that most residues on the binding surface of ACE2 are negative charge and polar, except K26 and K31 positive charge. The binding surface of SARS-CoV has one residue, D480, with a negative charge facing negative residues on ACE2, and most residues of SARS-CoV are polar and without charge. After evolution from SARS-CoV to SARS-CoV-2, the residue became negative E484 near the positive residue K31 of ACE2, and the T501N mutation increases the polarity. These mutations favor the interaction between SARS-CoV-2 and ACE2. All hydrogen bond (H-bond) interactions between the spike protein and ACE2 are listed in Fig. 3A, B. The distribution of H-bonds is different from SARS-CoV to SARS-CoV-2. For SARS-CoV, the residues on ACE2 involved in the H-bonds are K353, N330, Q325, Q42, Y41 and D38; for SARS-CoV-2, the residues on ACE2 involved in the H-bonds are Y83, Y41, H34, Q31 and Q24. This result indicated that the distribution of H-bonds shifted toward the N-terminus on SARS-CoV-2 compared with SARS-CoV. The change in the distribution of H-bonds is consistent with the influence of the residue 484 mutation from SARS-CoV to SARS-CoV-2 on the spike protein.

The evolution from SARS-CoV to SARS-CoV-2, and to Alpha variant and Delta variant enhanced the interaction of the spike protein with ACE2. In Fig. 3, the residues ID on binding interface of spike protein are labeled with red color and the arrows, which indicate the mutational residues on the binding interface of the spike protein of SARS-CoV-2. Alteration to charge residues is an important factor in virus evolution. SARS-CoV lacks an effective charge residue on the interface with ACE2. In contrast, SARS-CoV-2 has residue E484 with a negative charge near the positive K31 of ACE2. In Alpha variant, the E484K mutation reverses the charge of the residue from negative to positive and triggers a folding change, and K484 interacts with the nearby negative E23 on ACE2. In Delta variant, although the E484Q mutation changes from charged to polar, the N440K, L452R and T478K mutations changed to residues with positive charge. N440K changed from polar to positive charge and forwarded to negative residue E329 on ACE2; L452R changed from hydrophobic to positive charge and forwarded to negative residue D38 on ACE2; T478K changed from polar to positive charge and forwarded to negative residue E22 and E23 on ACE2. Also, it is noted that the K417N mutation (sub-lineages AY.1 and AY.2 in Delta variant) avoided the positive charge repulsion between K417 residue of spike protein and K31 residue of ACE2. These mutations in the Delta variant increase the affinity between the spike protein and ACE2. Overall, structural mutation analysis revealed that the evolution from SARS-CoV to SARS-CoV-2, and to Alpha variant and Delta variant enhanced the interactions of spike protein with ACE2, which enables the coronavirus to infiltrate into human cells and spread more easily.

## Discussion

The protein structure fingerprint is capable to align both sequence and structure conformation, and to reveal the changes in biologic function and folding conformation following mutations. In principle, sequence alignment is a useful means for studying mutations. First, it can handle a large amount of data from databases with multiple sequence alignments for residue-by-residue investigation. Second, the protein structure fingerprint provides a complete description of protein folding without any gap, generating a unique folding string for alignment to study the changes in folding conformation. With the protein structure fingerprint, the PFSC string as a complete folding description which is acquired according to either the protein 3D structure or the PFVM, can cover regular secondary fragments and irregular tertiary fragments. Third, the alignment of PFSC strings is able to discover the folding structure difference caused by mutation. In addition, the combination of alignment of sequence with PFSC alphabetic string provides comprehensive analysis for mutation investigation according residue by residue. Moreover, the PFVM as folding variations, which is obtained directly according to protein sequence, reveals the fluctuation of the folding conformation along the sequence. It is significant that the protein structure fingerprint overcomes barriers in the study of the effects of mutations when protein 3D structure data are absent. Thus, directly associating the protein sequence with the protein structure fingerprint is better to probe the mutations of SARS-CoV-2, which exhibits the changes on both its physiochemical properties and folding conformation, and provides more complete information for understanding the variations in biological functions caused by mutations.

The mutations in fragment at the binding interface of the RBD of the SARS-CoV-2 spike protein are critical for causing the coronavirus epidemic spread. Many researches has focused on the interaction between RBD of spike protein of SARS-CoV-2 and ACE2^28–33^. To interact with ACE2, the X-ray crystallography revealed that the SARS-CoV-2 RBD had a twisted five-stranded antiparallel β sheet (β1, β2, β3, β4 and β7) with short connecting helices and loops that form the core, which has four pairs of disulfide bonds to stabilize the structure^34^. Reversely, the ACE2 peptidase domain α1 helix is an important fragment for binding SARS-CoV-2-RBD. To compare SARS-CoV-2 RBD and ACE2 complex (PBD ID: 6M0J) and SARS-CoV RBD and ACE2 complex (PBD ID: 2AJF), with a distance cut-off of 4 Å, a total of 17 residues of the SARS-CoV-2-RBD are in contact with 20 residues of ACE2, while a total of 16 residues of the SARS-CoV-RBD are in contact with 20 residues of ACE2. However, the evolution from SARS-CoV to SARS-CoV-2 mutations increased 20-fold binding to ACE2^35^. The residues on contact interface for the SARS-CoV-2 RBD is located at region of T333–G526, and for the ACE2 at N-terminal peptidase domain of S19–D615. In our study, from SARS-CoV to SARS-CoV-2 (Fig. 3A, B), the binding interface at the RBD was involved with at least 9 residue mutations. Before SARS-CoV-2, the residues at the interface of SARS-CoV did not have apparent charge features, and most residues were polar. After evolution to SARS-CoV-2, residue E484 with a negative charge appears nearby positive charge residue K31 of ACE2, which is one of the factors making SARS-CoV-2 a more severe disease COVID-19 than SARS-CoV. The Alpha variant has 7 residue mutations (E484K, N501Y, A570D, P681H, T716I, S982A and D1118H) at the spike protein, but only E484K and N501Y are critical because of their positions at the binding interface of the RBD, which strengthen the interaction between SARS-CoV-2 and ACE2 to increase SARS-CoV-2 infectivity. The Delta variant has many mutations in the spike protein, but only K417N, N440K, L452R, T478K and E484Q directly impact the interaction with ACE2. Although the E484Q mutation reduced the charge feature of the residue, the N440K, L452R and T478K mutations generated three positive residues near the negative residues E329, D38, E22 and E23 in ACE2, and K417N mutation reduced the repulsion. Thus, the mutations in Delta variant enhanced the affinity between the spike protein and ACE2 and then increased the viral function. Furthermore, the Delta variant may become the sub-lineages AY.1 and AY.2 with the K417N mutation^36^, which raised concerns about the possibility of the reduced effectiveness of vaccines and antibody, and increased risk of reinfection^37^. With observation of alignment in Tables 1 and 2, the coronavirus evolved from residue V404 of SARS-CoV to residue K417 of SARS-CoV-2, which changed from hydrophobic to hydrophilic and from non-charge to positive charge. Additionally, the K417N mutation not only occurred on Delta AY.1 and AY.2 variants, but was also presented in the Beta (lineage B.1.351) and Gamma (lineage P.1) variants^38^. The K417N mutation changed from positive charge to polar property, which reduced the positive-positive charge repulsion between SARS-CoV-2 and ACE2 and may contribute to immune escape. Also, bioassay experiments showed that the K417N mutation certainly conferred > 100-fold reduced susceptibility to antibody etesevimab^39^ and about tenfold reduced susceptibility to antibody casirivimab^40^. Thus, the K417N mutation is needed to further study due to involving virus spread and drug development. As viruses undergo genetic changes, some of these genetic changes can confer evolutionary advantages, and mutations of SARS-CoV-2 at the binding interface with ACE2 are especially critical. In the process of evolution, many mutations occurred at different positions in the spike protein and even on other proteins of SARS-CoV-2^41^. Of course, some mutations may be neutral because they involve substitution of amino acids with physicochemical similarity; some mutations are missense because of substitution of amino acids with different physicochemical properties that change viral biological function. Thus, the protein structure fingerprint approach offers a useful means to align both sequence and complete folding conformation for investigation of the changes according residue by residue, which better expose structural variations in evolutions of SARS-CoV-2 and other viruses.

SARS-CoV-2 variant may be more transmissible than previously evolved ones, so understanding structural variations is important for development of antibodies and vaccines, novel protease inhibitors and repurposed drugs. The structural variations caused by mutations can provide leading information for vaccine and antibody research. With new mRNA vaccine technology, short-lived synthetic fragments of the RNA sequence of a virus is introduced into the human body, where they are taken up by dendritic cells, which use their own internal ribosomes to read the mRNA and produce viral antigen proteins. The synthetic mRNA fragment is a copy of the specific part of the viral RNA that carries the instructions to build the protein spike of SARS-CoV-2. Thus, the structural variations at the binding interface of the RBD of the SARS-CoV-2 spike protein provide an important reference for designing synthetic mRNA fragments. Developing a cocktail with multiple synthetic mRNA fragments according to the mutations in the fragment at binding interface of the RBD of the spike protein may be a solution to continuously counter the evolution of SARS-CoV-2. Moreover, antibody engineering requires structural data related to spike protein mutations to design therapeutic product appropriately. Antibodies contain complementarity determining regions (CDRs) for a particular epitope on specific antigen, allowing these two structures to bind together with precision. Mutations in the RBD of the SARS-CoV-2 spike protein provide significant structural information for CDR design and production of effective antibodies. Thus, understanding the structural variations, particularly at the RBD of the spike protein of SARS-CoV-2, is substantial for vaccine and antibody development.

## Conclusion

The alignment of both protein sequence and folding description reveals the structural variations caused by mutations of SARS-CoV-2. The protein structure fingerprint applies an alphabetical string to achieve a complete description of folding, which provides supplemental structural information for mutation study. The integration of changes in both physicochemical properties and folding features at the affinity interface of the RBD of the spike protein revealed how the coronavirus has gradually evolved in both structure and function and why SARS-CoV-2, Alpha variant and Delta variant have led to more severe acute disease worldwide.

## Methods

### Structural bioinformation

All protein structural data for SARS-CoV-2 were extracted from public databases. The sequences were obtained from the NCBI and UniProt databases, and protein 3D structures were obtained from the PDB. The cd21477 cluster was obtained from the NCBI Conserved Domain Database with LOCUS: APO40579, which contains the protein structure with PDB ID 6ACC for the SARS-CoV spike protein released in August 2018 and the protein structure with PDB ID 6VSB for the SARS-CoV-2 spike protein released in February 2020. Then, the mutations between 6ACC and 6VSB were analyzed according to either protein 3D structures or sequences by protein structure fingerprint technology. Information on the Alpha variant (lineage B.1.1.7) and Delta variant (lineage B.1.617.2) of SARS-CoV-2 was obtained from Public Health England. The information of AY.1 and AY.2 sub-lineage variants was obtained from Outbreak.info Website and Stanford University Coronavirus Antiviral & Resistance Database. Seven mutations in the spike protein were identified, and the variations in physiochemical properties and folding conformations were studied. The complexes of coronavirus with ACE2 were obtained from PDB, which PBD ID 6ACG is the complex with SARS-CoV and PBD ID 7A98 is the complex with SARS-CoV-2.

### Protein comparison

The sequences of the spike protein between SARS-CoV and SARS-CoV-2 were aligned with the Clustal Omega program through UniProt and then compared according to their physiochemical properties. Discovery Studio (version 4.5) was used to generate 3D images of protein structures, and then the superimposition of protein 3D structures was performed. Furthermore, with protein structure fingerprint technology, the variations in protein folding conformations were exposed in detail.

### Protein structure fingerprint

**Figure 4:**
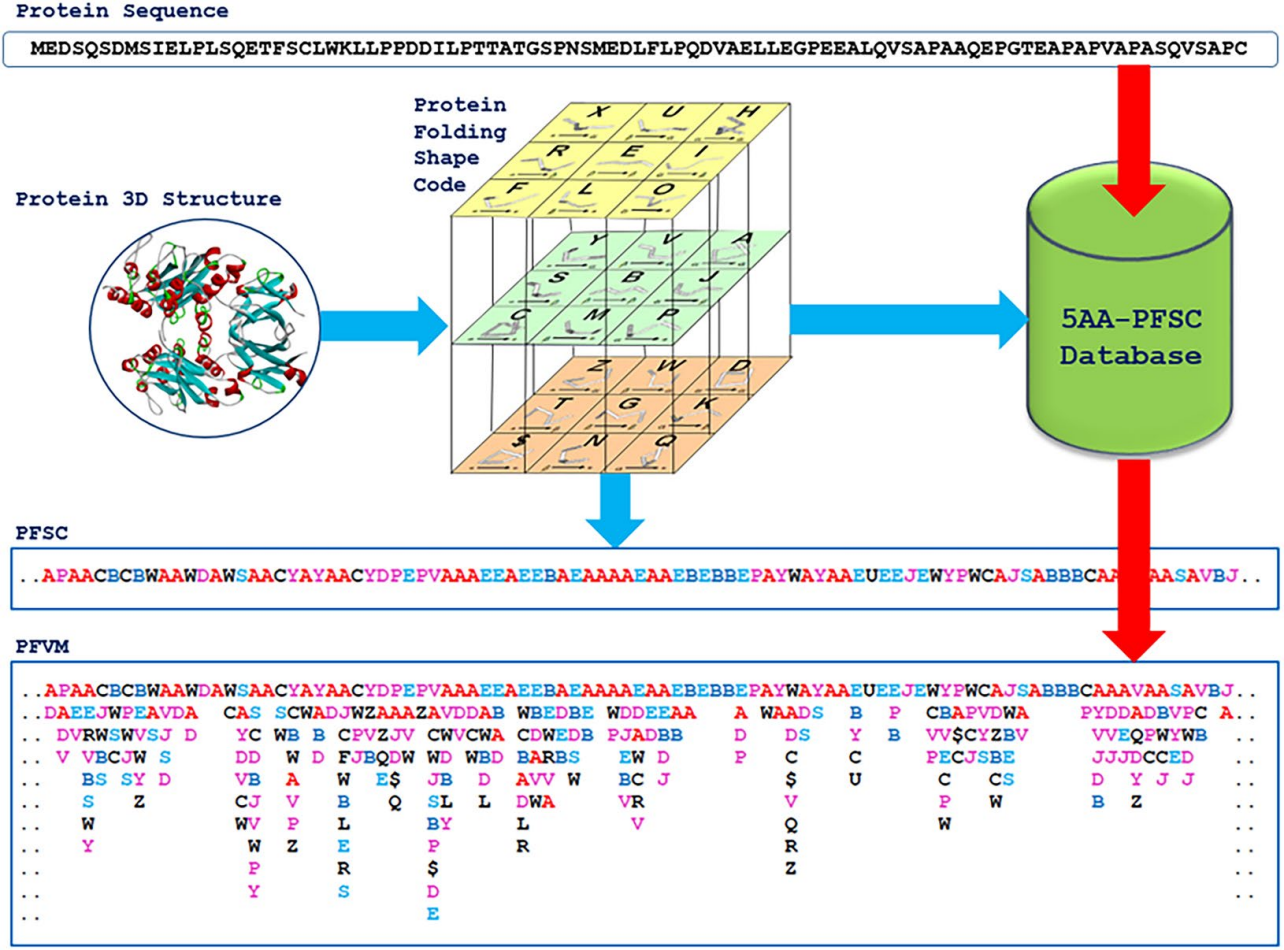
Protein structure fingerprint technology. The set of 27 protein folding shape code (PFSC) is presented in the cubic box. The blue arrows indicate how the complete conformation description with using PFSC is obtained from a protein 3D structure. The red arrows indicate how the comprehensive protein folding variations in the protein folding variation matrix (PFVM) are obtained from protein sequence, and expressed in PFSC description.

First, the complete folding space for a set of 5 successive points was mathematically covered by a set of folding shapes. Second, the possible folds of a fragment of 5 amino acids could be defined by the 27 protein folding shape code (PFSC) with alphabetical letters, as shown in Fig. 4. Third, any protein sequence has a protein folding variation matrix (PFVM), and any protein with a given 3D structure can be expressed by a PFSC string as a protein structure conformation. In the PFSC string, two PFSC letters next each other overlap by four amino acids; thus, a PFSC string represents the complete folding conformation of the 3D protein structure. It is significant that protein folding conformations with PFSC strings or PFVM can be aligned for comparison protein structures^42–44^. Therefore, the folding variations of SARS-CoV-2 as well as its mutations may be well analyzed by protein structure fingerprints.

### Software availability

The protein structure fingerprint can be accessed on Website http://www.micropht.com.
